# Bifidobacterium as a Potential Biomarker of Sarcopenia in Elderly Women

**DOI:** 10.3390/nu15051266

**Published:** 2023-03-03

**Authors:** Zhengyuan Wang, Xin Xu, Yangzong Deji, Shanxi Gao, Chunxiang Wu, Qi Song, Zehuan Shi, Xuesong Xiang, Jiajie Zang, Jin Su

**Affiliations:** 1 Division of Health Risk Factors Monitoring and Control, Shanghai Municipal Center for Disease Control and Prevention, Shanghai 200336, China; 2 School of Public Health, Key Lab of Public Health Safety of the Ministry of Education, Fudan University, Shanghai 200032, China; 3 Division of Health Risk Factors Monitoring and Control, Shanghai Municipal Fengxian District Center for Disease Control and Prevention, Shanghai 200336, China; 4 Division of Chronic Disease Control, Shanghai Municipal Putuo District Center for Disease Control and Prevention, Shanghai 200336, China; 5 Element Nutrition of National Health Commission, National Institute of Nutrition and Health China Center for Disease Control and Prevention, Beijing 100050, China

**Keywords:** bifidobacterium, sarcopenia, gut microbiota, elderly women

## Abstract

Gut microbial dysbiosis influences the development of sarcopenia. This case-control study explored the gut microbiota composition in elderly Chinese women with sarcopenia. The information from 50 cases and 50 controls was collected. Grip strength, body weight, body mass index, skeletal muscle mass, energy intake, and total and high-quality protein intake were lower in cases than in controls (*p* < 0.05). Gut microbiota metagenomic sequencing showed that phylum *Bacteroides* was significantly reduced in the case group, whereas genus *Prevotella* was more abundant (*p* < 0.05). Linear discriminant analysis (LDA) effect size showed that 9 and 13 distinct microbial taxa were enriched in the case and control groups, respectively (LDA > 2, *p* < 0.05), among which *Prevotella copri* and *Bifidobacterium longum* were significantly different (LDA > 4, *p* < 0.05). The AUC of *Bifidobacterium longum* was 0.674 (95% CI: 0.539–0.756). Elderly women with sarcopenia exhibited significantly different gut microbiota compositions than healthy controls.

## 1. Introduction

Sarcopenia is a common geriatric syndrome characterized by progressive and widespread loss of muscle mass and function, which may lead to an increased risk of adverse health events such as falls, fractures, disability, and death [1]. With the rapid growth of the aging population, the prevalence of sarcopenia has also been rising. The incidence of sarcopenia varies from 10% to 25% in different countries because of ethnicity and different diagnostic criteria [2]. Notably, in some regions, the incidence of sarcopenia can be as high as 50% in the elderly aged 80–89 years [3]. A cross-sectional study conducted at Sichuan University recruited 4224 subjects over the age of 50 years from western China, of whom 19.3% were diagnosed with sarcopenia [4]. According to a study conducted in Shanghai in 2018, the prevalence of sarcopenia among men and women older than 60 years in the community was 14.9% and 14.0%, respectively [5].

At present, there is no suitable clinical drug for sarcopenia, and nutrition and exercise are recognized to be effective. Previous intervention studies have yet to reach consistent treatment methods. However, some have suggested that the gut microbiota is an important intermediate link in the effects of dietary nutrition and exercise on sarcopenia [6]. Recently, the interaction between the skeletal muscle mass and gut microbiota (muscle-gut axis) has received attention. According to several systematic reviews, aging affects muscles and causes dysbiosis of the gut microbiota. Meanwhile, changes in gut microbiota diversity and composition and decreased metabolites of beneficial bacteria will further promote chronic inflammation and anabolic resistance, ultimately leading to reduced muscle size, impaired muscle function, and adverse clinical outcomes [7,8]. Evidence from a number of animal experiments supports a causal link between restoring a functional gut microbiota and improving muscle function [9,10], suggesting that targeting the gut microbiota could be a potential strategy to combat sarcopenia.

Currently, there is a paucity of studies on bacterial markers. Several case-control studies found differences in microbiota composition between sarcopenic patients and controls, but the bacteria strains reported were different. On the one hand, this may be due to the influence of different countries, dietary habits, geography, and biochemical modalities on the taxonomic composition of the gut microbiota [11]. On the other hand, due to the different hormonal levels and physiological characteristics between men and women, the gut microbiota characteristics are also different [12], and the ratio of men to women in the included subjects may affect the results of differential strains of bacteria. In addition, since the risk of muscle loss after age 50 is 20 times higher for women than men [13], there is a need to assess the relationship between gut microbiota and sarcopenia in older women. Therefore, in the present case-control study, we focused on elderly women to explore the effect of gut microbiota on the occurrence of sarcopenia in the first quarter of 2021.

## 2. Materials and Methods

### 2.1. Participants

This community-based case-control study recruited 50 individuals with sarcopenia and 50 healthy controls from Huangpu District and Putuo District. Women aged 65–75 years, able to walk independently, living in the community for more than 2 years, and voluntarily participating in this study were included. It was ensured that the participants had not taken antibiotics, probiotics, or other drugs that affect gut microbiota 48 h prior to the collection of fecal samples. Individuals were excluded if they had cognitive dysfunction, diseases of the heart, liver, kidney, or other important organs, hip and knee pain and dysfunction, a recent history of trauma, fracture, or surgery, or other diseases that rendered them unfit or unable to exercise, or if they were taking drugs that interfered with the study (e.g., drugs for thyroid function).

### 2.2. Screening Criteria

The clinical diagnosis method for sarcopenia from the consensus updated by the European Working Group on Sarcopenia in Older People (EWGSOP) in 2018 and the criteria from the consensus updated by the Asian Working Group on Sarcopenia (AWGS) in 2019 were used to screen the sarcopenia population [1,14] stepwise as follows:

The elderly women aged 65–75 years in the community were screened using the SARC-F questionnaire recommended by the EWGSOP, and the criteria recommended by the AWGS were used to further assess the individuals with a score ≥4.

If a participant’s pace was <1.0 m/s in the pace test, their muscle mass was assessed; if their pace was ≥1.0 m/s, their hand grip strength was assessed.

Sarcopenia was ruled out if the grip strength of the dominant hand was normal at rest (≥18 kg for females); if hand grip strength was lower than normal, muscle mass was assessed.

Bioimpedance analysis was used to quantify skeletal muscle mass. Sarcopenia was ruled out if skeletal muscle/height2 ≥ 5.7 kg/m^2^ (for females); if skeletal muscle/height2 < 5.7 kg/m^2^ (for females), the participant was diagnosed with sarcopenia.

### 2.3. Data and Biological Sample Collection

The data were collected using questionnaires. General information questionnaires consisted of age, gender, education level, marital status, pre-retirement occupation, and smoking status. The food frequency questionnaire was a validated and reliable questionnaire with 15 food categories and 117 items to assess the frequency and average food intake over the previous three months [15]. Quality protein was calculated based on the protein content of soy, milk and dairy products, meat and poultry, fish and seafood, eggs, preserved animal foods, snacks, and dietary supplements. A physical examination was conducted, including measurements of height, weight, grip strength, and body composition. Body composition, including body fat percentage, limb and trunk muscle mass, and waist-to-hip ratio, was assessed through bioimpedance analysis using INBODY 770 analyzers (Biospace, Seoul, Republic of Korea). We calculated BMI and grouped it based on the Chinese Guidelines for the Prevention and Control of Overweight and Obesity in Adults. We also used disease history surveys. The participants answered the questionnaires one-on-one with trained investigators.

Furthermore, soybean-sized fecal samples for gut microbiota detection were collected in test tubes with a stabilizing solution, transported to the laboratory within 48 h, and stored at −80 °C.

### 2.4. Gut Microbiota Metagenomic Detection

**Figure 1:**
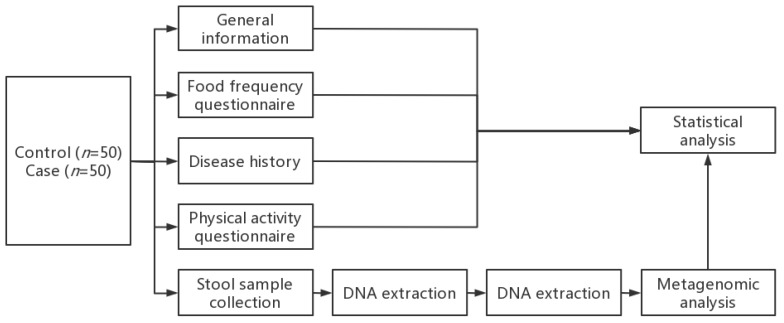
Research flow design chart.

Beijing QuantiHealth Technology Co., Ltd. provided assistance for gut microbiota metagenomic detection. Fecal bacterial DNA was extracted using the DNeasy PowerSoil Pro Kit (Qiagen, Germantown, MD, USA) according to the manufacturer’s instructions. Total DNA quality and quantity were estimated using spectrophotometry and 1% agarose gel electrophoresis, respectively. Then, DNA libraries were constructed using KAPA HyperPlus PCR-free library kits and sequenced on the Illumina Novaseg 6000 platform following the manufacturer’s instructions. The research flow design chart is as follows (Figure 1):

### 2.5. Statistical Analysis

The relative abundance of gut microbiota at various taxonomic levels was predicted using MetaPhlAn 2.0 [16] using clean reads, and permutational analysis of variance (PERMANOVA) was performed to test the difference between groups. The nonparametric Wilcoxon rank-sum test and Kruskal-Wallis test were used to analyze alpha indexes and taxonomic abundance. Linear discriminant analysis (LDA) effect size (LEfSe) [17] of the relative abundance of gut microbiota between control and case groups were performed to identify the potential biomarkers of sarcopenia. Taxa with LDA values > 2 with a *p*-value < 0.05 were considered significantly enriched. Strains with LDA values > 4 were those with significant differences in abundance between groups. Furthermore, the area under the curve (AUC) and the receiver operating characteristic curve (ROC) were used to assess the predictive value of significantly different strains between groups as bacterial markers for elderly female sarcopenia patients. A Spearman correlation analysis was used to analyze the correlation between gut microbiota and skeletal muscle mass, exercise habits, quality protein intake, BMI, and education level. Multivariate linear correlation analysis was used to analyze the linear relationship between the abundance of bacterial taxa and sarcopenia, skeletal muscle mass, BMI, education level, exercise habits, and quality protein intake.

Data other than biological samples were analyzed using SPSS 26.0. Normally distributed data were presented as mean ± standard deviation, and an independent sample t-test was used for comparison between groups. Nonnormally distributed data were expressed as medians and quartiles, and a nonparametric test was used for comparisons between groups. Enumeration data were analyzed using the chi-square test. A logistic regression analysis was used to investigate the factors that influence sarcopenia in elderly female patients. *p* < 0.05 was considered to indicate statistical significance.

## 3. Results

### 3.1. Baseline Characteristics

The basic characteristics of the participants included age, educational level, marital status, occupation type before retirement, smoking status, height, weight, body mass index (BMI), grip strength, and average daily activity level. The average grip strength, body weight, and BMI in the case group were significantly lower than those in the control group (*p* < 0.05). There were significant differences in the composition of educational level between the two groups (*p* < 0.05). Other characteristics were not significantly different between the two groups (Table 1).

### 3.2. Dietary Intake

Differences in energy intake and regular nutrient intake between the two groups were analyzed. The energy, protein, and high-quality protein intakes of the case group were significantly lower than those of the control group (*p* < 0.05). There were no significant differences in the intake of fats, calcium, vitamin D, soybean products, dairy products, vegetables, fruits, poultry, meat, and fish between the two groups (Table 2).

### 3.3. Body Composition

The two groups showed significant differences in skeletal muscle mass and the skeletal muscle mass/weight, skeletal muscle mass/BMI, and skeletal muscle mass/body fat percentage ratios (*p* < 0.001). There was no significant difference in body fat percentage between the two groups (Table 3).

### 3.4. Bacterial Composition Analysis

**Figure 2:**
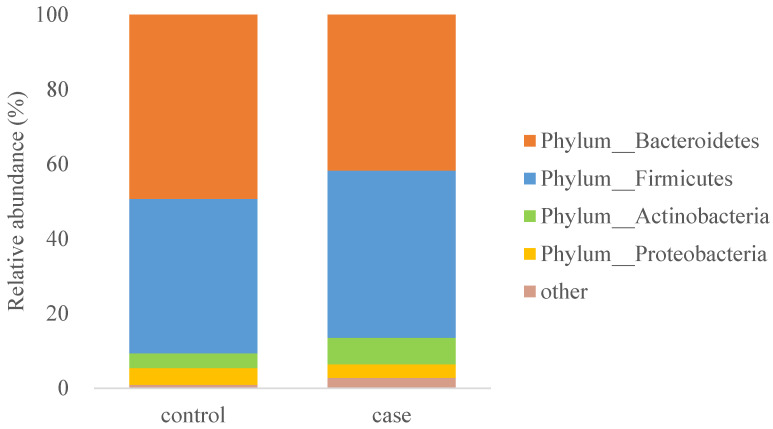
Relative abundance of various bacterial phyla.

A comparison of the relative abundance of gut microbiota at the phylum level showed significant differences in the gut microbiota composition between the two groups (*p* < 0.05). More than 97% of the gut microbiota in the two groups could be classified into four dominant bacterial phyla, namely *Bacteroidetes*, *Firmicutes*, *Actinomycetes*, and *Proteobacteria*. These four bacterial phyla accounted for 41.71%, 44.75%, 7.13%, and 3.60%, respectively, in the case group and 49.30%, 41.32%, 3.98%, and 4.45%, respectively, in the control group (Figure 2).

### 3.5. Linear Discriminant Analysis Effect Size Analysis

**Figure 3:**
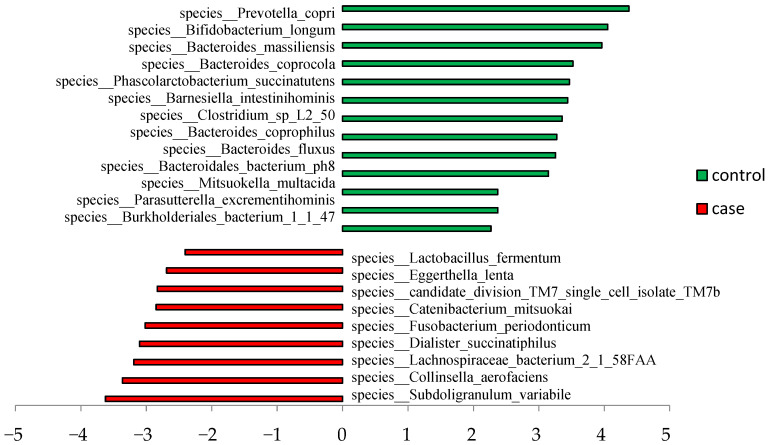
Linear discriminant analysis (LDA) score (log 10).

Significantly abundant taxa were further analyzed as categorical biomarkers of sarcopenia. LefSe analysis demonstrated that 9 and 13 distinct microbial taxa were significantly enriched in the case and control groups, respectively (LDA > 2, *p* < 0.05). *Prevotella copri* and *Bifidobacterium longum* showed significantly different abundances between the case and control groups and could be considered potential taxonomic biomarkers of sarcopenia at the species level (LDA > 4, *p* < 0.05; Figure 3).

### 3.6. ROC Analysis

**Figure 4:**
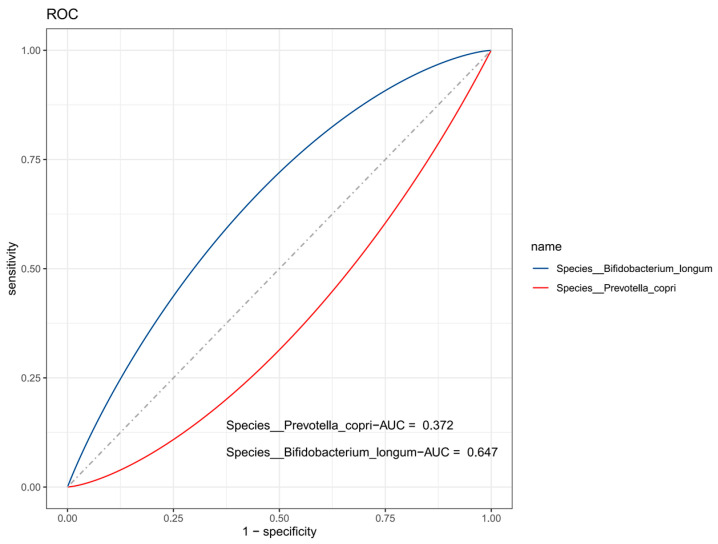
ROC curves for differential strain assessment of sarcopenia in older women.

The sensitivity of *Bifidobacterium longum* in predicting sarcopenia in older women was 53.1%, and the specificity was 74.0% (AUC = 0.647, 95% CI: 0.539–0.756). The sensitivity of blood *Prevotella copri* in predicting sarcopenia in older women was 4.1%, and the specificity was 98.0% (AUC = 0.372, 95% CI: 0.261–0.484) (Table 4; Figure 4).

### 3.7. Spearman Correlation Analysis

**Figure 5:**
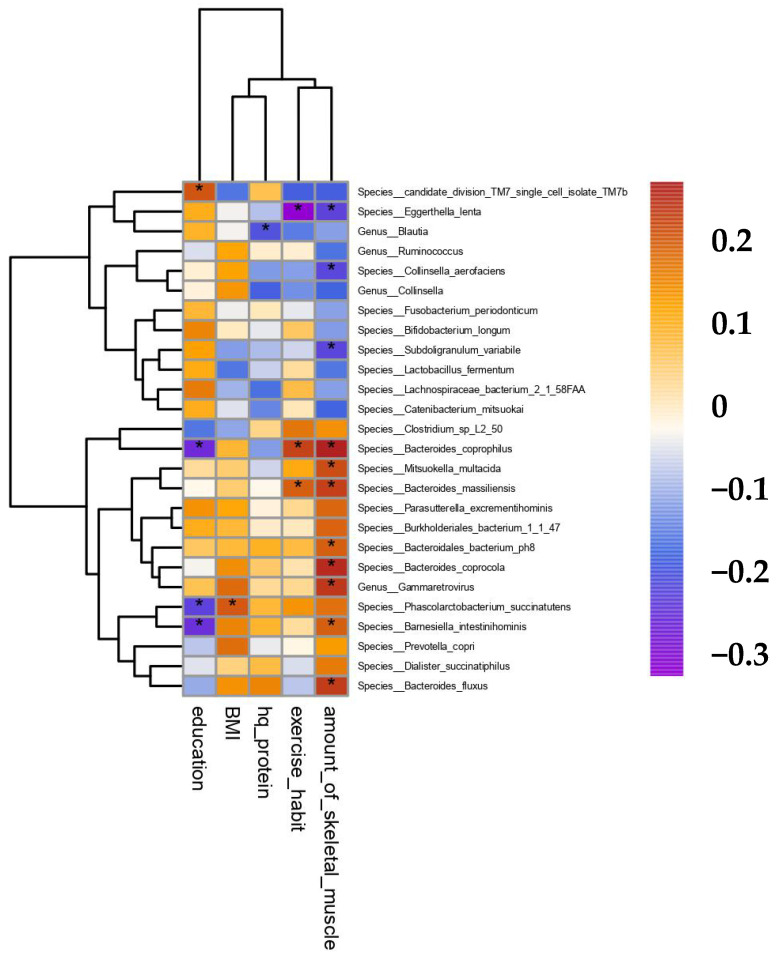
Spearman’s correlation heatmap. * indicates *p* < 0.05.

The correlations between different gut microbiota and skeletal muscle mass, exercise habits, high-quality protein intake, BMI, and educational level were analyzed separately. The results showed that the abundance of *Bacteroides fluxus*, *Barnesiella intestinihominis*, *Bacteroides coprocola*, *Bacteroidales bacterium (ph8)*, *Bacteroides massiliensis*, *Mitsuokella multacida*, and *Bacteroides coprophilus* at the species level and Gammaretrovirus at the genus level was positively correlated with skeletal muscle mass (*p* < 0.05). The abundance of *Eggerthella lenta*, *Collinsella aerofaciens*, and *Subdoligranulum variabile* and skeletal muscle mass were negatively correlated (*p* < 0.05; Figure 5).

### 3.8. Multivariate Linear Correlation Analysis

A multivariate linear correlation analysis was performed with sarcopenia, skeletal muscle mass, BMI, educational level, exercise habits, and high-quality protein intake as dependent variables and sarcopenia, skeletal muscle mass, BMI, educational level, exercise habits, and high-quality protein intake as independent variables. At the strain level, *p. copri (GCF000157935)* and *Bifidobacterium bifidum* exhibited statistically significant differences between the two groups (*p* < 0.05). At the genus level, *Atopobium* exhibited a statistically significant difference between the two groups (*p* < 0.05; Table 5).

## 4. Discussion

Aging causes a gradual imbalance in human muscle synthesis and metabolism, which is closely associated with the development of sarcopenia [18]. The incidence of sarcopenia is increasing annually as the global aging process accelerates. In recent years, potential pathways linking the gut with muscle have been discovered [19], and studies on the gut-muscle axis suggest that the composition of the gut microbiota is associated with sarcopenia [7]. Previous studies lacked data on the Chinese population. Considering that gender differences may influence the gut microbiota distribution and that women are a high-risk group for sarcopenia, we conducted a case-control study on elderly women in a Shanghai community. The results showed that the energy intake, high-quality protein intake, and BMI were lower in individuals with sarcopenia than in the control group. The gut microbiota metagenomics analysis also revealed differences in the composition of the gut microbiota between the two groups, which may be responsible for the functional differences.

We found low energy, total protein, and exceptionally high-quality protein intake in the case group. Although we did not find a strong correlation between nutritional status and multiple single strains of bacteria, previous studies have pointed out that prebiotics, fermented foods, energy, fat, protein, and carbohydrates can modulate the composition of the gut microbiota [20]. Indeed, we found differences in the gut microbiota of elderly women with sarcopenia and controls. The proportion of the phylum *Actinobacteria* in the case group was approximately 79% higher than that in controls. LEfSe analysis showed that *Collinsella aerofaciens* and *Eggerthella lenta*, species belonging to the phylum Actinobacteria, were significantly enriched in the case group, whereas *Bifidobacterium longum* was the second most highly enriched in the control group, with an LDA score of approximately 4.05. This finding suggests that various taxa belonging to *Actinobacteria* have different effects on sarcopenia.

We also figured out that the AUC of *Bifidobacterium longum* for predicting sarcopenia in older women was 0.647, which is more accurate than other indicators. *Bifidobacterium longum* may be a beneficial species for sarcopenia, as demonstrated in several animal experiments and human trials conducted in other countries. A clinical trial in which *Bifidobacterium longum* was orally administered to mice for 12 weeks reported that *Bifidobacterium longum* improved the gastrocnemius and tibialis muscles and enhanced muscle functions such as grip strength and physical endurance [21]. A 12-week clinical intervention trial in patients with sarcopenia in Japan also demonstrated an association between increased *Bifidobacterium longum* and recovery from muscle atrophy [22]. *Bifidobacterium longum* may affect sarcopenia through the action of its metabolites, such as short-chain fatty acids (acetate, propionate, and butyrate), which are central to regulating immune and metabolic homeostasis [23]. *Bifidobacterium longum* also contributes to the absorption and utilization of vitamin D and minerals such as calcium, phosphorus, and iron [24], which are important for muscle metabolism. Moreover, multivariate analysis showed that *Bifidobacterium bifidum* was a protective factor for sarcopenia, which may be associated with the function of the microbial genome. A joint study by Ohio State University and Benha University revealed that the *Bifidobacterium bifidum* genome is rich in genes encoding proteins involved in carbohydrate and amino acid transport and metabolism, affecting protein synthesis and nutrient utilization [25], which are closely associated with the prevention and treatment of sarcopenia. As a result, *Bifidobacterium* may be important gut bacteria for sarcopenia treatment and relief.

In the present study, multiple analyses showed an association between *Bacteroides* and sarcopenia. At the phylum level, *Bacteroidetes* showed a higher abundance in the control group than in the case group. LEfSe analysis indicated that species belonging to the *Bacteroidetes*, including *bacterium ph8*, *Bacteroides fluxus*, *Bacteroides coprophilus*, *Barnesiella intestinihominis*, *Bacteroides coprocola*, and *Bacteroides massiliensis*, were significantly different between the two groups. Univariate analysis found that *Bacteroides* species (e.g., *Bacteroides fluxus*, *Bacteroides coprophilus*, *Bacteroides coprocola*, and *Bacteroides massiliensis*) were significantly positively associated with skeletal muscle mass. A previous review highlighted that *Bacteroides* are involved in many important metabolic activities, including fermentation of carbohydrates, utilization of nitrogenous substances, and biotransformation of steroids [26], which may explain the positive correlation between *Bacteroides* and skeletal muscle mass. *Bacteroides* are also the main synthesizers of vitamin K [27]. A cross-sectional survey in Japan showed vitamin K plays a very important role in regulating bones and body calcium, which increases bone mineral density and improves exercise capacity [28]. Few studies have focused on the effect of *Bacteroides* species on sarcopenia, which requires further investigation.

At the phylum level, the abundance of *Firmicutes* was higher in the case group, and the *Firmicutes*/*Bacteroidetes* ratio (F/B ratio) in the case group was approximately 30% higher than that in the control group. The F/B ratio is also widely considered to be important for maintaining normal intestinal homeostasis, and a decrease in the ratio is often observed in inflammatory diseases [29]. An inflammatory environment accelerates protein catabolism and induces sarcopenia. A prospective cohort study in Italy that recruited 332 elderly people aged 80 years and older reported that the levels of pro-inflammatory factors such as interleukin-6, tumor necrosis factor-α, and C-reactive protein were 2–4 times higher in patients with sarcopenia than in nonsarcopenic participants [30]. These findings suggest that maintaining the F/B ratio is critical and that it may serve as a monitoring indicator of inflammatory levels in patients with sarcopenia.

Linear correlation analysis found that *p. copri (GCF000157935)* was positively associated with sarcopenia. LEfSe analysis showed that *Prevotella copri* was significantly enriched in the control group. However, the AUC value of *Prevotella copri* was 0.372, indicating that although *Prevotella copri* differed between the two groups, it was insufficient to be used as a single biomarker for predicting sarcopenia. Currently, there is no international consensus on the relationship between *Prevotella* and sarcopenia. A review of intestinal permeability by German scholars suggests that a marked increase in *Prevotella* indicates the evolution of a mucin-degrading niche and may lead to epithelial barrier disruption, increased intestinal permeability, and the induction of inflammation, thereby affecting protein anabolism [31]. However, other studies suggest that *Prevotella* is associated with muscle synthesis. A case-control study in the United States showed that older adults with high muscle strength had higher fecal levels of *Prevotella* than controls [32]. These inconsistent findings indicate that different species or strains of *Prevotella* have different effects; therefore, the effect of *Prevotella* on sarcopenia needs to be further explored.

There are relatively few clinical studies on the effect of gut microbiota on sarcopenia in China, and our study provides more data on the association between gut microbiota composition and sarcopenia in older women. The present case-control study focused on older women, which eliminated the effect of gender differences. However, our study also has certain limitations. We have focused on differences in gut flora composition in female patients, but the applicability of these findings to other populations still needs to be further explored. Nutritional status affects the composition of the gut microbiota, and the mediating effects of the gut microbiota still need to be further explored. Although *Bifidobacterium longum* is superior to *Prevotella copri* as a predictor of sarcopenia in older women and possibly a potential target, its accuracy is not high, and more clinical trials are needed to validate it. Moreover, the collection of participant information in case–control trials may be associated with recall bias.

## 5. Conclusions

Elderly women with sarcopenia had poorer nutritional status than controls, manifested in lower energy intake, protein intake, and BMI. There are also differences in gastrointestinal microbiomes between the two groups. *Bifidobacterium*, especially *Bifidobacterium longum*, may have a protective effect on elderly women with sarcopenia. It is suggested that intervention studies can be carried out in this population. In addition, the effect of *Prevotella copri* on sarcopenia is controversial. People with sarcopenia are advised to pay attention to changes in the gastrointestinal microbiota and intervene as early as possible.

## Figures and Tables

**Table 1:** Basic characteristics of study participants in each group.

Variable	Control	Case	F/X^2^	*p*
Age, years	68.7 ± 3.4	68.4 ± 3.5	0.19	0.661
Educational level, *n* (%)			7.42	0.024
High school and above	25 (50)	12 (24)		
Junior high school	22 (44)	32 (64)		
Primary school and illiterate	3 (6)	6 (12)		
Marital status, *n* (%)			0.64	0.424
Married	43 (86)	40 (80)		
Divorced or widowed	7 (14)	10 (20)		
Pre-retirement occupation, *n* (%)			0.19	0.656
Mental work mainly	37 (74)	35 (70)		
Physical labor mainly	13 (26)	15 (30)		
Smoking, *n*(%)			0.34	0.558
Smoker	1 (2)	2 (4)		
Nonsmoker	49 (98)	48 (96)		
Height, cm	157.8 ± 4.9	156.8 ± 5.2	1.08	0.302
Weight, kg	60.0 ± 6.3	55.5 ± 6.7	11.99	0.001
BMI, kg/m^2^	24.1 ± 2.4	22.5 ± 2.3	10.49	0.002
BMI group			3.87	0.145
Underweight	1 (2)	3 (6)		
Normal	24 (48)	31 (62)		
Overweight	25 (50)	16 (32)		
Grip strength, kg	20.9 ± 1.2	16.3 ± 1.8	233.34	<0.001
Average daily duration of moderate physical activity, median (P25,P75)	0.0 (0.0, 30.0)	0.0 (0.0, 5.4)	2.47	0.119

**Table 2:** Comparison of dietary intake between the control and case groups.

Variable	Control	Case	F/X^2^	*p*
Energy, kJ	8334.3 ± 2732.0	7095.5 ± 3234.7	4.28	0.041
Total protein, g	88.9 ± 34.6	74.3 ± 36.9	4.18	0.044
High-quality protein, g	53.1 ± 26.4	40 ± 23.5	6.81	0.010
Fat, g	57.2 ± 22.5	53.3 ± 30.0	0.54	0.464
Ca, mg	846.7 ± 502.8	691.7 ± 446.2	2.66	0.106
Vitamin D, IU	1.1 ± 6.0	1.1 ± 3.9	0.01	0.990
Soybean and its products, g	9.1 (4.9, 20.9)	9.3 (5.0, 14.4)	0.524	0.600
Milk and its products, g	240.0 (113.9, 303.2)	180.9 (0.0, 5.4)	0.790	0.430
Vegetable, g	225.9 (123.3, 333.3)	205.8 (147.0 319.5)	0.059	0.953
Fruit, g	124.4 (76.7, 226.3)	96.5 (53.5, 236.9)	0.900	0.368
Poultry and meat, g	63.8 (41.2, 103.8)	43.0 (24.1, 82.1)	1.958	0.050
Fish, g	41.7 (27.1, 64.4)	37.2 (24.3, 95.9)	0.407	0.684

**Table 3:** Comparison of dietary intake between the control and case groups.

Variable	Control	Case	F/X^2^	*p*
Skeletal muscle mass, kg	16.9 ± 1.8	13.5 ± 1.3	120.03	<0.001
Body fat percentage, %	35.8 ± 5.6	35.3 ± 5.4	0.22	0.640
Skeletal muscle mass/weight	0.30; ± 0.02	0.24 ± 0.02	8.11	<0.001
Skeletal muscle mass/BMI	0.71 ± 0.08	0.60 ± 0.08	44.32	<0.001
Skeletal muscle mass/body fat percentage	0.49 ± 0.1	0.40 ± 0.08	27.26	<0.001

**Table 4:** Results of ROC analysis.

Variable	AUC	95% CI	Cutoff	Youden Index	Sensitivity	Specificity
Species__Bifidobacterium_longum	0.647	0.539–0.756	0.19	0.271	0.531	0.740
Species__Prevotella_copri	0.372	0.261–0.484	0.024	0.021	0.041	0.980

**Table 5:** Results of multiple linear analyses of bacterial flora.

Reference Group	Feature	Coefficient	*p*
Control	Strain__Prevotella_copri_GCF_000157935	0.126	0.001
Control	Strain__Bifidobacterium_bifidum_unclassified	−0.148	0.020
Control	Genus__Atopobium	−0.019	0.027

## Data Availability

Not applicable.

## References

[1] Cruz-Jentoft A.J., Bahat G., Bauer J., Boirie Y., Bruyère O., Cederholm T., Cooper C., Landi F., Rolland Y., Sayer A.A. (2019). Sarcopenia: Revised European consensus on definition and diagnosis. Age Ageing.

[2] Cruz-Jentoft A.J., Landi F., Schneider S.M., Zúñiga C., Arai H., Boirie Y., Chen L.K., Fielding R.A., Martin F.C., Michel J.P. (2014). Prevalence of and interventions for sarcopenia in ageing adults: A systematic review. Report of the International Sarcopenia Initiative (EWGSOP and IWGS). Age Ageing.

[3] Chen Z., Ho M., Chau P.H. (2021). Prevalence, Incidence, and Associated Factors of Possible Sarcopenia in Community-Dwelling Chinese Older Adults: A Pop-ulation-Based Longitudinal Study. Front. Med..

[4] Zhao W.Y., Zhang Y., Hou L.S., Xia X. (2021). The association between systemic inflammatory markers and sarcopenia: Results from the West China Health and Aging Trend Study (WCHAT). Arch. Gerontol. Geriatrics.

[5] Zhang Y., Chen X., Hou L., Lin X., Qin D., Wang H., Hai S., Cao L., Dong B. (2020). Prevalence and Risk Factors Governing the Loss of Muscle Function in Elderly Sarcopenia Patients: A longitudinal Study in China with 4 Years of Follow-Up. J. Nutr. Health Aging.

[6] Strasser B., Wolters M., Weyh C., Krüger K., Ticinesi A. (2021). The Effects of Lifestyle and Diet on Gut Microbiota Composition, Inflammation and Muscle Performance in Our Aging Society. Nutrients.

[7] Liu C., Cheung W.H., Li J., Chow S.K.H., Yu J., Wong S.H., Ip M., Sung J.J.Y., Wong R.M.Y. (2021). Understanding the gut microbiota and sarcopenia: A systematic review. J. Cachexia Sarcopenia Muscle.

[8] Zhang T., Cheng J.K., Hu Y.M. (2022). Gut microbiota as a promising therapeutic target for age-related sarcopenia. Ageing Res. Rev..

[9] Chen L.H., Chang S.S., Chang H.Y., Wu C.H., Pan C.H., Chang C.C., Chan C.H., Huang H.Y. (2022). Probiotic supplementation attenuates age-related sarcopenia via the gut-muscle axis in SAMP8 mice. J. Cachexia Sarcopenia Muscle.

[10] Giron M., Thomas M., Jarzaguet M., Mayeur C., Ferrere G., Noordine M.L., Bornes S., Dardevet D., Chassard C., Auzeloux I.S. (2022). Lacticaseibacillus casei CNCM I-5663 supplementation maintained muscle mass in a model of frail rodents. Front. Nutr..

[11] Deschasaux M., Bouter K.E., Prodan A., Levin E., Groen A.K., Herrema H., Tremaroli V., Bakker G.J., Attaye I., Sietsma S.J.P. (2018). Depicting the composition of gut microbiota in a population with varied ethnic origins but shared geography. Nat. Med..

[12] Yoon K., Kim N. (2021). Roles of Sex Hormones and Gender in the Gut Microbiota. J. Neurogastroenterol. Motil..

[13] Yang L., Smith L., Hamer M. (2019). Gender-specific risk factors for incident sarcopenia: 8-year follow-up of the English longitudinal study of ageing. J. Epidemiol. Community Health.

[14] Chen L.K., Woo J., Assantachai P., Auyeung T.W., Chou M.Y., Iijima K., Jang H.C., Kang L., Kim M., Kim S. (2020). Asian Working Group for Sarcopenia: 2019 Consensus Update on Sarcopenia Diagnosis and Treatment. J. Am. Med. Dir. Assoc..

[15] Song J., Zang J., Tang H., Li W., Wang Z., Zou S., Jia X. (2016). Relative validity of food frequency questionnaire for estimating dietary nutrients intake. Wei Sheng Yan Jiu.

[16] Segata N., Waldron L., Ballarini A., Narasimhan V., Jousson O., Huttenhower C. (2012). Metagenomic microbial community profiling using unique clade-specific marker genes. Nat. Methods.

[17] Segata N., Izard J., Waldron L., Gevers D., Miropolsky L., Garrett W.S., Huttenhower C. (2011). Metagenomic biomarker discovery and explanation. Genome Biol..

[18] Cruz-Jentoft A.J., Sayer A.A. (2019). Sarcopenia. Lancet.

[19] De Sire R., Rizzatti G., Ingravalle F., Pizzoferrato M., Petito V., Lopetuso L., Graziani C., Sire A., Mentella M.C., Mele M.C. (2018). Skeletal muscle-gut axis: Emerging mechanisms of sarcopenia for intestinal and extra intestinal diseases. Minerva Gastroenterol. Dietol..

[20] Zhang X., Shi L., Li Q., Song C., Han N., Yan T., Zhang L., Ren D., Zhao Y., Yang X. (2022). Caloric Restriction, Friend or Foe: Effects on Metabolic Status in Association with the Intestinal Microbiome and Metabolome. J. Agric. Food Chem..

[21] Ni Y., Yang X., Zheng L., Wang Z., Wu L., Jiang J., Yang T., Ma L., Fu Z. (2019). Lactobacillus and Bifidobacterium Improves Physiological Function and Cognitive Ability in Aged Mice by the Regulation of Gut Microbiota. Mol. Nutr. Food Res..

[22] Tominaga K., Tsuchiya A., Nakano O., Kuroki Y., Oka K., Minemura A., Matsumoto A., Takahashi M., Kadota Y., Tochio T. (2021). Increase in muscle mass associated with the prebiotic effects of 1-kestose in super-elderly patients with sarcopenia. Biosci. Microbiota Food Health.

[23] Wang I.K., Wu Y.Y., Yang Y.F., Ting I.W., Lin C.C., Yen T.H., Chen J.H., Wang C.H., Huang C.C., Lin H.C. (2015). The effect of probiotics on serum levels of cytokine and endotoxin in peritoneal dialysis patients: A randomised, double-blind, placebo-controlled trial. Benef. Microbes.

[24] Montazeri-Najafabady N., Ghasemi Y., Dabbaghmanesh M.H., Talezadeh P., Koohpeyma F., Gholami A. (2019). Supportive Role of Probiotic Strains in Protecting Rats from Ovariectomy-Induced Cortical Bone Loss. Probiotics. Probiotics Antimicrob. Proteins.

[25] Abdelhamid A.G., El-Dougdoug N.K. (2021). Comparative genomics of the gut commensal Bifidobacterium bifidum reveals adaptation to carbohydrate utilization. Biochem. Biophys. Res. Commun..

[26] Zafar H., Saier M.J. (2021). Gut bacteroides species in health and disease. Gut Microbes.

[27] Walther B., Karl J.P., Booth S.L., Boyaval P. (2013). Menaquinones, bacteria, and the food supply: The relevance of dairy and fermented food products to vitamin K requirements. Adv. Nutr..

[28] Fujita Y., Iki M., Tamaki J., Kouda K., Yura A., Kadowaki E., Sato Y., Moon J.S., Tomioka K., Okamoto N. (2012). Association between vitamin K intake from fermented soybeans, natto, and bone mineral density in elderly Japanese men: The Fujiwara-kyo Osteoporosis Risk in Men (FORMEN) study. Osteoporos. Int..

[29] Walker A.W., Sanderson J.D., Churcher C., Parkes G. (2011). High-throughput clone library analysis of the mucosa-associated microbiota reveals dysbiosis and differences between inflamed and non-inflamed regions of the intestine in inflammatory bowel disease. BMC Microbiol..

[30] Landi F., Calvani R., Lorenzi M., Martone A.M., Tosato M., Drey M., Angelo E.D., Capoluongo E., Russo A., Bernabei R. (2016). Serum levels of C-terminal agrin fragment (CAF) are associated with sarcopenia in older multimorbid community-dwellers: Results from the ilSIRENTE study. Exp. Gerontol..

[31] Bischoff S.C., Barbara G., Buurman W., Ockhuizen T., Schulzke J.D., Serino M., Tilg H., Watson A., Wells J.M. (2014). Intestinal permeability--a new target for disease prevention and therapy. BMC Gastroenterol..

[32] Fielding R.A., Reeves A.R., Jasuja R., Liu C., Barrett B.B., Lustgarten M.S. (2019). Muscle strength is increased in mice that are colonized with microbiota from high-functioning older adults. Exp. Gerontol..

